# Supporting contaminated sites management with Multiple Criteria Decision Analysis: Demonstration of a regulation-consistent approach

**DOI:** 10.1016/j.jclepro.2021.128347

**Published:** 2021-09-20

**Authors:** Marco Cinelli, Michael A. Gonzalez, Robert Ford, John McKernan, Salvatore Corrente, Miłosz Kadziński, Roman Słowiński

**Affiliations:** a Institute of Computing Science, Poznan University of Technology, Piotrowo 2, 60-965, Poznan, Poland; b Environmental Decision Analytics Branch - Land Remediation and Technology Division, Center for Environmental Solutions and Emergency Response, Office of Research and Development, U.S. Environmental Protection Agency, Cincinnati, 45268, Ohio, USA; c Contaminated Sites and Sediments Branch - Land Remediation and Technology Division, Center for Environmental Solutions and Emergency Response, Office of Research and Development, U.S. Environmental Protection Agency, Cincinnati, 45268, Ohio, USA; d Emerging Contaminants and Technologies Branch - Land Remediation and Technology Division, Center for Environmental Solutions and Emergency Response, Office of Research and Development, U.S. Environmental Protection Agency, Cincinnati, 45268, Ohio, USA; e Department of Economics and Business, University of Catania, Corso Italia, 55, 95129, Italy; f Systems Research Institute, Polish Academy of Sciences, Newelska 6, 01-447, Warsaw, Poland

**Keywords:** Site remediation, Multiple criteria decision analysis, Impact assessment, Environmental management, Decision support

## Abstract

This study proposes a set of key decision-making features of the contaminated site remediation process to assist in selecting the most appropriate decision support method(s). Using a case study consistent with the requirements of the U.S. regulation for contaminated sites management, this article shows that suitable Multiple Criteria Decision Analysis methods can be selected based on a dynamic and evolving problem structuring. The selected methods belong to the family of PROMETHEE methods and can provide ranking recommendations of the considered alternatives using variable structures of the criteria, evaluation of the alternatives and exploitation of the preference model. It was found that in order to support a quick and up-to-date application of powerful decision support techniques in the process of remediation of contaminated sites, decision analysts and stakeholders should interact and co-develop the process. This research also displays how such interactions can guarantee a transparent and traceable decision recommendation so that stakeholders can better understand why some alternatives perform comprehensively better than others when a multitude of inputs is used in the decision-making process.

## 1. Introduction

Remediation of contaminated sites is a complex process that requires a consideration of multiple sources of information, evaluation criteria, preferences, and trade-offs. Multiple Criteria Decision Analysis (MCDA) (Greco et al., 2016) is a rich methodology that has been developed over several last decades to account for multiple inputs of complex decision-making challenges explicitly, and the remediation of contaminated sites is undoubtedly one of them (Grelk et al., 1998; Havranek, 2019). MCDA components include alternatives, evaluation criteria, and preferences, which are used to formally structure and solve decision-making problems. One of its main potentials consists in accounting for conflicting criteria and non-dominating alternatives to find the most relevant compromise solution(s), according to stakeholders’ preferences (Costa et al., 2018; Rocchi et al., 2019). There are several individual applications of MCDA methods for ranking alternatives that could be used to remediate contaminated sites. For example, Bates et al. (2016) used Multi-Attribute Value Theory (MAVT) (Keeney and Raiffa, 1976) to rank emerging technologies for environmental remediation by comparing nanotechnology and synthetic biology to conventional remediation methods. The main criteria used in this study included risks, benefits, and costs. Bates et al. (2014) chose PROMETHEE (Brans and Vincke, 1985; Behzadian et al., 2010) as the MCDA method to rank remediation alternatives in the Norwegian fjords, including uncertainty modelling on the input performances. Nasiri et al. (2007) ranked groundwater remediation alternatives using a flexible MCDA method that accepts uncertain data in terms of linguistic judgments and experts’ opinions. Todaro et al. (2021) developed a preference index to rank different reactive capping designs based on criteria accounting for their life cycle implications. Several reviews are available of MCDA methods and tools used for environmental remediation, including Huysegoms and Cappuyns (2017), Kiker et al. (2005), Linkov et al. (2006a), Cegan et al. (2017), and Onwubuya et al. (2009).

Adaptive site management is also proposed as a complement to the remediation process. This management approach is specifically focused on including the significant uncertainties inherent to the development of remediation plans, among which remedy performances and interests of multiple stakeholders (Price et al., 2017). MCDA has also been very successful in including these uncertainties in environmental remediation (Hokkanen et al., 2000; Sparrevik et al., 2012; An et al., 2016), as well as in other areas, like coastal zones management (Félix et al., 2012), infrastructure planning (Zheng et al., 2016) and energy production (Prado et al., 2020). This makes MCDA a clear candidate to aid the comprehensive assessment of alternatives in these kinds of application areas.

The common use of MCDA methods for the remediation of contaminated sites starts with the problem description and proceeds to the selection of the MCDA method(s) and provision of the results for each case study. In this application area, there is no identified approach yet to explicitly account for the features of these decision-making problems and justify the selection of some suitable MCDA method(s). This is the main knowledge gap that this contribution begins filling. This research implements the recent approach provided by Cinelli et al. (2020), which suggests the alignment between an explicit description of the decision-making problem with some chosen MCDA method(s).

In this paper, we advance the approach for selecting the relevant MCDA method according to some decision-making features used to describe the remediation alternatives assessment. To achieve this, we use a hypothetical case study to demonstrate the proposed approach and apply the recommended MCDA method(s) to aid in the comprehensive assessment of available alternatives. The generalizability of the proposed approach is lastly described to show that it is applicable to any other plan for the management of contaminated sites. This paper aims to increase the acceptance and application of MCDA in the area of contaminated site remediation by showing how the different requirements and preferences from stakeholders can drive the choice of suitable MCDA method(s). The MCDA method recommendations are based on an analysis of the challenges that several experts in the remediation process of contaminated sites experience in their daily work. The application area for this work is the Comprehensive Environmental Response, Compensation and Liability Act (CERCLA), informally called Superfund, which mandates the US Environmental Protection Agency (EPA) to remediate contaminated sites (EPA, 2015a). The Superfund cleanup process is complex and challenging and requires consideration that includes an aggregation of types and quantities of information (e.g., performance, costs, risks, benefits). To manage the amount and type of information, MCDA is an excellent approach for handing diverse information to support decision-making (Havranek, 2019).

Even if MCDA applications specifically for Superfund sites have already been published (Kyle Satterstrom et al., 2007; Yatsalo et al., 2007; Linkov et al., 2021), they have not explored the decision-making requirements that the respective regulation allows defining to steer the selection of the MCDA methods. An example is the metadata included in the detailed evaluation reports that study the feasibility of the alternatives and provide multiple possible structures of the evaluation criteria and their evaluation. In addition, no explicit interaction with the stakeholders involved in the Superfund process has been performed to co-constructively identify the capabilities that the MCDA methods should have to derive the decision recommendation. This paper fills these research gaps by developing a bridge between the Superfund regulation and the MCDA domain using the direct input of several U.S. EPA employees involved in the implementation of this regulation.

Let us mention that the integration of adaptive management and MCDA has already been discussed in the previous literature, e.g., in Foran et al. (2015) and Linkov et al. (2006b). The novelty of our research consists in embedding in the decision support processes we developed the recent recommendation from the U.S. EPA Superfund Task Force (EPA, 2018), which invites the development of dynamic and updatable strategies to manage decision uncertainty. We achieve this goal by shaping different MCDA processes according to the available information and requirements from the stakeholders. All these processes are always matched with the formal requisites of the Superfund regulation, including the set of criteria that has to be used to evaluate the alternatives. The latter is another aspect not matched in previous literature.

The paper is organized as follows: Section 2 presents the contribution that MCDA can have in the part of the Superfund cleanup process. Section 3 offers the material and methods used to frame the selection and application of the MCDA methods to assess remediation alternatives. Section 4 describes the results of the case study. Section5 discusses the main findings, and Section 6 concludes the paper.

## 2. Potential role of MCDA in the superfund cleanup process

MCDA is a methodology that allows for developing a comprehensive comparison of alternatives characterized by performances on multiple evaluation criteria (Roy, 1996; Neves et al., 2018; Cinelli et al., 2020). Its power resides in its capacity to convey a wealth of information representing each alternative in the process of, for example, ranking them from “best” to “worst” or in the process of their classification (e.g., into good, medium, and bad) (Ibáñez-Forés et al., 2014). MCDA is comprised of three components: alternatives, evaluation criteria, and preferences. Alternatives are the objects that represent potential actions that are evaluated from multiple points of view, criteria represent these points of view (i.e., the manners) on the quality of the alternatives, and preferences exhibit value systems of the stakeholders and ensure that the comprehensive comparison of alternatives is consistent with them. In MCDA, the alternatives are discrete, which means a finite set of alternatives is assessed by a pool of criteria. In most MCDA problems, there are non-dominating alternatives, meaning that no alternative performs at least as well as the others on all criteria and better for at least one of them. Consequently, the alternative(s) that can be recommended are those that represent the best compromise according to the considered criteria. Stakeholder preferences in the decision-making process can be included to consider the different priorities on the criteria and the aggregation of performances on these criteria.

The Superfund cleanup process includes decision-making challenges that can be managed with MCDA. The ones we focus on are the remedial investigation/feasibility study and the initial remedy selection of the Superfund cleanup process. These include a detailed analysis of the remediation technology alternatives that can be used to bring the selected contaminants to the desired concentration level. This assessment of individual alternatives has to be based on nine evaluation criteria, including threshold, balancing, and modifying criteria, as listed in Table 1 (EPA, 2015b).

Superfund regulation requires that those alternatives that do not satisfy the threshold criteria are eliminated from any further assessment (EPA, 2015b). Consequently, the evaluation of the remaining alternatives is based on the balancing and modifying criteria. The latter criteria are used to informally discuss the proposed alternatives and potentially modify the existing set of alternatives or extend it. The remedial investigation/feasibility study of the Superfund cleanup process can thus lead to an information matrix composed of *X* alternatives and the five balancing criteria, scored qualitatively (e.g., low, moderate, better) and quantitatively (e.g., $) (EA-EST, 2011). However, the main question that emerges at this stage is, “Which alternative performs comprehensively better than the others to be recommended as a preferred alternative, while also accounting for potential different criteria priorities?”. MCDA has specific methods that can contribute to solving this challenge. This paper focuses on such contribution, showing how the evaluation of remedial alternatives can be supported by a set of MCDA methods that comprehensively account for their performance and provide a preferential ranking.

## 3. Material and methods

In a recent report, the U.S. EPA Superfund Task Force recommended the development of dynamic work strategies to optimize data collection for the assessment of the alternatves (EPA, 2019). To support the achievement of this goal, as part of the efforts led by the Environmental Decision Analytics Branch in the Office of Research and Development (ORD) of the US EPA, three meetings were held in 2019 and 2020 with several EPA employees to obtain a better understanding of the main challenges faced during the Superfund cleanup process’s remediation alternatives prioritization. The attendees to these meetings represent a wide pool of expertise and skills in managing the remediation of contaminated sites, with a specific focus on the Superfund regulation. These employees (stakeholders in MCDA terminology) contributed their experiences to understanding the context, limitations, and main challenges faced during the Superfund cleanup remediation alternatives prioritization process. The full list of stakeholders is presented in Appendix 1 in the Electronic Supplementary Information (ESI).

The meetings led to a list of specific research questions (RQ) that are recurrent issues to be faced when supporting decision-making during the remedial investigation/feasibility study and the initial remedy selection of the Superfund cleanup process. They include:

- RQ 1: Which alternative, based on the data in the performance matrix and the chosen criteria priorities, performs comprehensively better than the others to be recommended as a preferred alternative based on the five balancing criteria?
- RQ 2: How to account for the sub-criteria (metadata) used to define and measure the balancing criteria?
- RQ 3: How to account for uncertainties in the input data and its measurements of the individual criteria and sub-criteria?
- RQ 4: How to account for a null level of compensation between the criteria and/or the sub-criteria?
- RQ 5: How to include preferences concerning the importance of the criteria and/or the sub-criteria used to derive their (an alternative’s) overall value?

The subsequent section discusses how combinations of the RQs were addressed with different MCDA methods.

### 3.1. The selected MCDA methods

To support the Superfund cleanup process, different MCDA processes and strategies can be developed into final recommendations for a decision on the considered alternatives (EPA, 1988a, 2018). As part of this project, different MCDA methods were selected and used to support the identification of a suitable preferred alternative. Based on the input received from the three meetings with the US EPA employees, four different MCDA processes were shaped and are shown in Table 2. In what follows, we refer to the processes by their numbers presented in Table 2. Each MCDA process tackles the identified RQs. The selection of the recognized MCDA methods was driven by a set of features related to the RQs and used to describe each MCDA process. These were selected from a recent taxonomy of the MCDA process by Cinelli et al. (2020) and include:

1. *The type of the desired outcome (linked to RQ 1)*: This consists of the kind of desired decision recommendation that must be provided by the MCDA method. In this project, it is always a complete ranking of the alternatives, driven by a score.
2. *The structure of the criteria (linked to RQ 2)*: This defines the organization of the criteria used for the assessment. It can be either flat (i.e., the criteria are all at the same level) or hierarchical (i.e., the criteria are organized in levels).
3. *Evaluation of alternatives on the criteria (linked to RQ 3):* This defines the type of data used in the assessment. In the simplest case, they are all expressed on an ordinal scale. Costs can also be included in their original measurement scale (i.e., $). Also, the evaluations can be either deterministic or uncertain. Experience has shown that there are a number of scenarios in which professional judgement is used as a substitute for time-intensive analysis of causal factors. For example, a scenario that is not uncommon is the collection of analytical data from site characterization reported with analytical limits that exceed what is commonly desired. The outcome is uncertainty in the value of the reported data. Since these data underpin decisions, their quality may influence the decision path. In the absence of a repeat of a data collection effort to improve analytical metrics, the uncertainty from these data may be accounted for with the modelling structure of MCDA methods.
4. *Compensation level between (sub-)criteria (linked to RQ 4)*: This considers how the good performance on one (sub-)criterion can compensate for the poor performance on another (sub-)criterion. In this project, the compensation level is null, as the considered criteria cannot compensate between each other.
5. *Weights of the criteria (linked to RQ 5):* They can be used to express the importance of the criteria. In this case study, they are interpreted as importance coefficients and considered as equal in all the processes.
6. *Analysis of robustness (linked to RQ 3 and 5):* This looks at how variable the recommendation can be when there is variability with respect to the performances of alternatives and/or stakeholders’ preference. In this project, only the former type of uncertainty is considered.

The requirements of deriving a complete ranking associated with scores, the need to account for a flat as well as a hierarchical structure of criteria, the lack of compensation between criteria, and the required capacity to include variable input data with a stochastic characterization of the results, have led us to the selection of the family of PROMETHEE methods. In these methods, the alternatives are compared pairwise to evaluate whether one is preferred to the other. Depending on the criteria structure, uncertainty related to the performances of alternatives and criteria weights, we selected suitable variants of PROMETHEE II (see Table 2). In particular, since the case dealing with a hierarchical structure of criteria was required, we used the Multiple Criteria Hierarchy Process (Corrente et al., 2013). Furthermore, when some performances were uncertain, we opted to conduct a robustness analysis with the Stochastic Multicriteria Acceptability Analysis (SMAA (Lahdelma et al., 1998); driven by the Monte Carlo simulations (Corrente et al., 2014). The methodological details on the selected PROMETHEE-based methods are provided in Appendix 2 in the ESI. In addition to these objective selection requirements, a driving factor was also the communicability to the U.S. EPA experts of the working strategy of this family of outranking methods. What is more, the last requirement was a consistent use of the methods, since changing the family of MCDA methods for each process would have added a notable challenge in terms of explanation about how the decision recommendations are provided in each process.

### 3.2. Case study description

The main recommendation that emerged from meetings with the US EPA experts involved in the Superfund cleanup process was to select a hypothetical case study to develop MCDA processes. The study choice was an extension of a previous case study presented in EPA (1988b), which is a guidance document on the implementation of Superfund regulation. The first main reason for the selection of this case study is that the research is a proof of concept, and its applicability is independent of any Superfund site, making the proposed approach and methods suitable for any contaminated site. The second reason for this case study choice is to develop a decision analysis framework that directly aligns with the structure of existing Superfund guidance, which applies five balancing criteria for evaluation of remediation alternatives (EPA, 2015b). In fact, current FSs of Superfund sites summarize the performance of the remediation technologies using the five balancing criteria (e.g., Tables 4.3–3 in EPA (2016) and Tables 5–1in AECOM (2019)). Further details on the reasons behind the choice of this hypothetical case study are presented in Appendix 3 of the ESI.

The summary from the detailed analysis of the case study in EPA (1988b) is provided in Table 3 and was used as the source for the two datasets employed in this study. These include a flat and hierarchical structure of the five balancing criteria, shown in Table 4 and Table 5, respectively. Table 4 provides a comprehensive score for each balancing criterion, like the summary tables presented in the FS of Superfund sites (e.g., EPA (2016) and AECOM (2019)). Table 5 uses the same information in Table 3 but, in this case, each piece of information is used to define multiple sub-criteria for each balancing criterion, except the cost one. This leads to a much more elaborate information table with the four remaining balancing criteria, now characterized by 13 sub-criteria.

It is important to note that the scoring provided in Tables 4 and 5, with the exception of the cost (*c*_5_), was derived from the interpretation of the descriptive language of the detailed analysis for the hypothetical case study presented in Table A-7 in Appendix A of EPA (1988b). It should also be noted that the analysis of the performance of the three remedial alternatives presented in this hypothetical case study is unique to site-specific conditions; performance characteristics may improve/-degrade under different site conditions. This exercise intends to illustrate the flexibility and adaptability of the MCDA process to address both the main, overarching criteria, as well as sub-criteria that may have specific importance in the decision process for a particular site. Ultimately, the sub-criteria can be adapted to address specific stakeholder perspectives or technical factors that may be linked to properties unique to the contaminant or physical characteristics of the site.

The process used to assign the qualitative scores consisted in judging the verbal description reported in Table 3. As an example, from the flat criteria structure in Table 3, criterion 2 “Reduction of toxicity, mobility, or volume through treatment” (*c*_2_) for Alternative 1 reads as “No treatment; no destruction; no reduction of MTV; residual contamination is high.” This verbal description can be comprehensively interpreted as a negative performance of the alternative, since it does not result in any reduction of toxicity, mobility or volume as no treatment is applied, and furthermore, the contamination that remains in the site is high, implying that it is not the desired outcome. Consequently, the assigned score to this criterion is 0 (assuming the higher the score, the better). This criterion provides two main pieces of information. Firstly, it indicates whether there is any treatment at the site, assuming there would be a reduction of toxicity, mobility, or volume. Secondly, it reports the residual contamination level. As part of the hierarchical structure of the criteria (Table 5), these pieces of information are used to define the two sub-criteria, treatment of contaminants (*sc*_3_) and residual contamination (*sc*_4_). A similar approach is also used for the other criteria, using the information provided for each one to define a global score in Table 4 and individual scores for the sub-criteria in Table 5.

Let us emphasize an important methodological consideration. The switch from a flat to a hierarchical structure of criteria does not only entail a different organization of the information. In fact, this choice actually implies that different criteria are considered under the same concepts, which may lead to different results. Regarding the weight of the criteria, all upper-level criteria (i.e., *c*_1−5_) are assigned the same weight (i.e., 0.2), which is then allocated according to the sub-criteria in the case of the hierarchically structured problems.

There are currently no guidelines to convert the verbal descriptions provided in the detailed analysis of alternatives (like in Table 3 in this case study) into the numerical input data presented in Tables 4 and 5. This was one of the key reasons why uncertainty modelling was adopted to account for the subjectivity that characterizes this semi-quantitative step. MCDA processes 3 and 4 include this strategy, which consists in using uncertainty modelling for the qualitative criteria (*c*_1−4_) based on the precautionary principle. This means that an assumption of one worse value (e.g., assuming positive polarity, if the score was 2, it can also be 1) is used in this research for the ordinal criteria. In the case of costs (*c*_5_), the uncertainty is accounted for by the range of −30% ≤ deterministic value ≤+50%, a common approach used in the development of remedial alternatives. These input data are then exploited by means of stochastic sampling, which consists in choosing a large number of times (e.g., 1,000,000 times for MCDA process 3 and 10,000,000 times for MCDA process 4) the values of the alternatives, within the defined boundaries. For illustrative purposes, Table 6 shows an example of input used in the stochastic modelling for Alternative 2 in MCDA process 3, whose original input data is shown in the first row. It can be seen that in simulation 1, the alternative receives a worse value compared to the original one for *c*_1_, *c*_2_, and *c*_5_. In simulation 2, the worse scores are for *c*_1_ and *c*_4_, while in simulation 3, the performance is worse on *c*_2_, *c*_4_, while it is better on *c*_5_. The possible range of variability of the input data is shown in the last row of the table, with either a 2 or 3 assigned to *c*_1_−_4_, and the costs with the lower bound of 2,100,000 $ (i.e., −30% on the deterministic value) and an upper bound of 4,500,000 $ (i.e., +50% on the deterministic value).

## 4. Results

The results of this case study are presented in a sequential manner, starting from the ones based on the simplest MCDA processes (i.e., 1 and 2 in Section 4.1) followed by the more complex ones (i.e., 3 and 4 in Section 4.2).

### 4.1. Deterministic results: MCDA process 1–2

PROMETHEE works with an outranking algorithm whose intermediate result is the pairwise comparison matrix revealing the comprehensive preference degrees. In Table 7, we present such a matrix for MCDA process 1, when all criteria have equal weights. For example, with respect to Table 4, Alternative 1 performs better than Alternative 2 on two out of five criteria, and it thus receives *π*(*a*_1_*, a*_2_) = 0.4. Furthermore, Alternative 2 is better than Alternative 3 on a pair of criteria, the inverse relation holds only for a single criterion, and on the remaining two criteria, these two alternatives attain the same performances. For this reason, *π*(*a*_2_*, a*_3_) = 0.4 and *π*(*a*_3_*,a*_2_) = 0.2.

The main outcome of PROMETHEE is the net flow of the alternatives (see Table 8), which indicates the overall performance of each alternative when its outranking strengths and weakness are combined. Alternative 2 net flow is positive in both processes 1 and 2, while Alternative 3 shows a null net flow for process 1 and a slightly negative one (i.e., −0.07) for process 2. Alternative 1 always receives a negative net flow, confirming that it is the worst performer in this set of alternatives.

Based on the input information for the flat (process 1) and hierarchical (process 2) criteria structures, Alternative 2 receives the highest score and, consequently, the best rank when compared to the other alternatives. All the MCDA models confirm this finding, showing that even if different problem structures are defined, both the MCDA processes (1, 2) using deterministic input always recommend Alternative 2 as the best performer.

Based on the input information for the flat criteria structure (MCDA process 1), Alternative 2 receives the highest score and, consequently, the best rank when compared to the other alternatives. When integrating the dynamicity in structuring the criteria and sub-criteria set, their number changes from five to 14, leading to MCDA process 2. Even with this change, it can still be seen in Table 8 that the more elaborate structure of criteria does not change the ranking result. Thus, these MCDA models thus show that even if different problem structures are defined, both the MCDA processes (1, 2) using deterministic input always recommend Alternative 2 as the best performer.

### 4.2. Stochastic results: MCDA process 3 and 4

All the input used in MCDA processes 1 and 2 is deterministic, and the results are a single ranking driven by the net flow score. The performances for criteria 1 to 4 (*c*_1−4_) and sub-criteria 1 to 13 (*sc*_1−13_) are qualitative and assigned based on the verbal descriptions in the summary of the detailed analysis of alternatives in Table 3. MCDA process 3 and 4 consider the subjectivity induced by the lack of guidelines to convert these verbal descriptions into numbers. This is tackled by a further elaboration of the MCDA processes, using uncertainty modelling for the qualitative criteria (*c*_1−4_) in MCDA process 3 and sub-criteria (*sc*_1−13_) in MCDA process 4, as well as for the costs (sub-)criterion. Based on the precautionary principle, stochastic simulation is used by choosing a very large number of times (i.e., more than 100,000 times) the values of performances of the alternatives.

The results of such processes are reported with the *Rank Acceptability Index (RAI)* and the *Pairwise Winning Index (PWI)* (see Appendix 2 in the ESI for details on these measures). RAI provides a global perspective in the form of ranking frequency when accounting for the comparisons with all the alternatives. PWI offers a binary comparison between alternatives, showing how each alternative performs when compared to another one.

The SMAA-based results confirm that, in both cases of flat and hierarchical criteria (Table 9 and Table 10, respectively) structure, the best alternative in the wide majority (i.e., >90%) of the simulations is Alternative 2, followed by Alternative 3 and lastly by Alternative 1. The PWIs for the SMAA-based results with the flat structure of criteria (Table 9) indicate a unanimous (i.e., 100% of the simulations) outperformance of Alternatives 2 and 3 compared to Alternative 1. Similar results are also obtained in the SMAA-based results with a hierarchical structure of criteria (Table 10), even if the PWI for Alternatives 2 and 3 compared to Alternative 1 are not 100% anymore, but 99.78% and 83.52%, respectively. The relative improvement of Alternative 1 from flat to the hierarchical structure of criteria shows that the modified framework of the problem, resulting in the inclusion of sub-criteria, leads to a different and more detailed evaluation of the alternatives. This modelling structure allows to better use the metadata in the FS, while the SMAA-based model permits to account for uncertainties in the information used to assess the alternatives. The main findings (i.e., Alternative 2 is the best, followed by Alternatives 3 and 1) remain, however, the same, as confirmed by the expected ranking (Kadziński and Michalski, 2016) (right side in Tables 9 and 10).

The hierarchical structuring of the criteria, presented in Table 11, enables “zooming into” the results and making them more transparent and understandable. For example, even if at the global/comprehensive level Alternative 2 ranks first in more than 90% of the simulations, it is apparent that it is not always the best performer, specifically on criteria *c*_3−5_. Firstly, on short term effectiveness (*c*_3_), it ranks mostly at the second position, with more than 10% of the simulations placing it in the last rank. Secondly, when considering implementability (*c*_4_), it receives the first position only in 7.67% of the cases, with Alternative 1 ranking first in more than 94.19% of the cases. Thirdly, it scores robustly (i.e., 94.8%) in the second position as far as costs (*c*_5_) are considered, and it never reaches the first rank. Nonetheless, when compared to the remaining alternatives accounting for all the criteria and sub-criteria, it outperforms them in the wide majority of the simulations (i.e., 92.1%).

The modelling structure based on the hierarchical structure of the criteria confirms that the individual analysis of the performance on each criterion can better highlight the strengths and weaknesses of the alternatives. In the case of SMAA-based process 4, this finding is enhanced by the capability of considering the uncertainty in the modelling of the performances. Alternative 1 mostly outperforms the other alternatives on *c*_4_ and *c*_5_, reaching the first position in 94.2% and 100% of the simulations, respectively. On the contrary, it is always the worst on *c*_1_ and *c*_2_, and in *c*_3_ for more than 75% of the simulations.

## 5. Discussion

The main contribution of this work has been to show how to select a suitable MCDA method to conduct a comprehensive evaluation of the alternatives based on the remedial investigation/feasibility study unique to Superfund regulation. This results in a ranking of the alternatives which combines their strengths and weaknesses, showing its potentials in aiding the selection of a preferred one. This research confirms that the analysis process is not static, but dynamic with evolving decision- making features that drive the selection of the relevant MCDA method. The input received from the US EPA personnel involved in the Superfund cleanup process has shown how different decision-making features can vary within the same Superfund site or from one site to another, which has a clear impact on the suitable MCDA method to be used to perform a comprehensive analysis of alternatives.

The most straightforward MCDA process (i.e., 1) can be focused on the performance table to obtain a single ranking of the alternatives using only the five balancing criteria with the deterministic evaluation as input and a balanced preference on the weights of the criteria (i.e., equal weights). This can be seen as the “status quo” or a starting point in MCDA-based ranking from the remedial investigation/feasibility study. The process can then be modified by including the extensive metadata included in such study, which is a focal added value of this research. In fact, the qualitative scoring given to criteria 1–4 (i.e., *c*_1_ = long-term effectiveness and permanence, *c*_2_ = reduction in toxicity, mobility, or volume through treatment, *c*_3_ = short-term effectiveness, and *c*_4_ = implementability) is frequently driven by substantial work that can belong to different lines of evidence. Our research characterized these metadata in the form of sub-criteria of each balancing criterion,^1^ showing its use in processes 2 and 4.

Another fundamental decision-making feature is the type of evaluations used for the alternatives. The “status-quo” choice is to use deterministic values in the form of ordinal scores (for *c*_1−4_) and actual costs\figures for *c*_5_*c*_5_ (see processes 1 and 2). However, these values are subject to uncertainty, and as indicated in the recent US EPA report on Superfund (EPA, 2019), the uncertainty in the decision-making process should be accounted for as an important part of adaptive management plans. This request has been satisfied by including the uncertainty in the performance evaluation in processes 3 and 4. This modelling setting leads to probability-distributed results, whose main advantage is to study the robustness of the outcomes and to allow evaluating whether a trend can be identified. Furthermore, the outcome is not univocal, which shows that it is still the decision maker who must make a final choice, confirming the role of these methods as aiding tools to reach a decision.

A notable advantage that is provided by MCDA is that it can facilitate an iterative development of the decision process. This could be especially useful for situations in which stakeholder preference is invoked as a reason not to select an alternative that ranks highest in the MCDA process. This can happen when the application of MCDA methods might be considered redundant since the stakeholders/DMs think that they have holistic preferences that are “good enough” for the final decision. We argue that this selection bias can be controlled or at least minimized with the use of MCDA methods. We provide a dedicated reflection in Appendix 4 in the ESI. In this respect, one could, in fact focus on either adding additional criteria or modify existing criteria to address the stakeholder preference. Then, the assessment could be re-run to evaluate whether the selection is sensitive to changes to the MCDA process. If not, then there is a documented analysis for reference that may be more acceptable than outright rejection of the stakeholder preference.

One avenue to consider for future application of MCDA as part of the Superfund cleanup process could be sites in which a remedy optimization evaluation is conducted at the request of a regional office. The different optimization strategies that can be proposed and are based on multiple criteria (including remedy effectiveness, cost reduction, technical improvement, green remediation, redevelopment potential (Biggs et al., 2018)) can be subject to an MCDA evaluation. It is unlikely that one optimization strategy outperforms the others on all the criteria, meaning that some form of compromise has to be made to reach a decision recommendation, which is what MCDA methods have been developed for.

The findings presented in this paper are not confined to the Superfund cleanup process. In turn, they are applicable to any other environmental decision-making process that deals with the challenge of remediating contaminated sites. What has been proposed in this research is (i) a set of crucial decision-making features of the contaminated site remediation process that can help to steer the selection of the most appropriate MCDA methods, and to (ii) structure the analysis of metadata included in reports describing the performance of proposed remediation alternatives into useable information for MCDA methods that can perform a comprehensive analysis of the alternatives and provide a decision recommendation (e.g., ranking, sorting, choice). The decision-making features presented in this paper are relevant for most of the complex remediation processes. To extend the set of criteria as part of adaptive management strategies, green remediation features can also be considered, which can include the quantities of energy used, quantity and types of materials needed, and emissions generated by each proposed alternative (EPA, 2012).

This research also comes with a series of limitations. Firstly, only a relatively limited group of experts participated in the development of the research questions. A natural extension of this research could thus involve the inclusion of more experts to discuss whether other questions could be included. Secondly, the only stakeholders involved in the meetings to identify the research questions are U.S. EPA employees and the decision support stops at the stage of the detailed analysis of alternatives using the five balancing criteria. This research does not account for what happens after the results of the comprehensive assessment of alternatives are shown to the other stakeholders, such as the general public or governmental officers. This extension of research could allow including different priorities (i.e., weights) for the criteria as well as inclusion of further alternatives. In order to test this proposal, the approach could be tested in an existing Superfund site. Lastly, other MCDA methods could be suitable to tackle the decision-making challenges presented in this article. To study their suitability, an evaluation of a subset of MCDA methods could be structured with respect to the features identified in this research to support decision-making. The methods that would be capable of handling such features could then be applied to the case study.

## 6. Conclusions

This research has revealed how the Superfund cleanup process can be interpreted as a dynamic and evolving set of Multiple Criteria Decision Analysis (MCDA) processes. A class of important decision-making features that should be considered during the process of remediating contaminated sites has been proposed. These features have been shown to be pivotal in steering the choice of a decision analysis method that can provide comprehensive analyses of the considered alternatives. These analyses consist in accounting for the strengths and weaknesses that each alternative has when compared to the others, resulting in a preference-ordered ranking that condenses much information in a decision recommendation. It must be noted that this recommendation is transparent as it is possible to trace the final ranking back to the individual performance of the alternatives, allowing the models to be “glass boxes” instead of “black boxes”. The primary learning insight from this project is that interaction between decision analysts and experts in the process of remediation of contaminated sites is fundamental to ensuring that powerful advances in decision support are incorporated in remediation and technology assessment. This can result in the efficient and effective use of a vast amount of information embedded in the lengthy and comprehensive evaluation of the remediation alternatives.

## Supplementary Material

Supplementary Data

## Figures and Tables

**Table 1:** Feasibility Study (FS) individual alternatives nine evaluation criteria (adapted from EPA (2015b)).

**Threshold Criteria**	Overall Protection of Human Health and the Environment Compliance with Applicable or Relevant and Appropriate Requirements (ARARs)
**Balancing Criteria**	Long-term Effectiveness and Permanence Reduction in Toxicity, Mobility, or Volume through Treatment Short-term Effectiveness Implementability (technical and administrative) Cost
**Modifying Criteria**	State Acceptance Community Acceptance

**Table 2:** Different MCDA processes with addressed Research Questions (RQs), recommended and applied MCDA methods for remediation alternatives prioritization in this case study.

Decision-making features	MCDA Process 1 (RQ 1, 4, 5)	MCDA Process 2 (RQ 1, 2, 4, 5)	MCDA Process 3 (RQ 1, 3, 4, 5)	MCDA Process 4 (RQ 1, 2, 3, 4, 5)

1. Desired outcome	Complete ranking driven by a score			
2. Criteria structure	Flat structure of criteria	Hierarchical structure of criteria	Flat structure of criteria	Hierarchical structure of criteria
3. Evaluation of the alternatives on the criteria	Qualitative measurement scales for four criteria, assessed on a five-point scale, scoring from very low to very high. One criterion (i.e., costs) is measured on a quantitative scale ($).		Qualitative measurement scales, assessed on a five-point scale for four criteria (i.e., excluding costs), scoring from very low to very high. Uncertainty in evaluating the alternatives using a precautionary assumption of one worse performance (e.g., assuming positive polarity, if the score was 2, it can also be 1). One criterion (i.e., costs) is measured on a cardinal scale with uncertainty in the form of −30% ≤ deterministic value ≤ +50%.	
4. Compensation level between (sub-) criteria	Null			
5. Weights of the criteria	Equal weights			
6. Robustness analysis	No → Single recommendation		Yes → Stochastic characterization of trends	

**MCDA methods recommended and applied**	PROMETHEE II	PROMETHEE II for hierarchical criteria	SMAA-PROMETHEE II	SMAA-PROMETHEE II for hierarchical criteria

**Table 3:** Summary of the detailed analysis of alternatives presented in the hypothetical case study in Appendix A of EPA (1988b) used to develop the datasets for this case study.

	Balancing criteria				
	
Alternative	Long-term effectiveness and permanence	Reduction of toxicity, mobility or volume through treatment	Short-term effectiveness	Implementability	Cost
					
	*C*_1_	*C*_2_	*C*_3_	*C*_4_	*C*_5_

**Alternative 1** - Natural attenuation	Potential for exposure from residual contamination because institutional controls such as deed restrictions are not effective. The risk for carcinogens is at the high end of the protective risk range (2 × 10^_4^) and the HI is above 1.0.	No treatment; no destruction; no reduction of MTV; residual contamination is high.	Presents a higher risk to the community over the short-term; does not cause exposure to workers; does not cause environmental impacts; the restoration time frame is 40 years.	Deed restrictions are unreliable; ease of taking additional actions is high; ability to monitor is high; ability to obtain approvals from other agencies is high; no coordination problem.	500,000 $
**Alternative 2** - Pulsed pumping, 3 well points, air-stripping, enhanced biodegradation	Residual risk is 10^_ 5^ for carcinogens, and the HI for systemic toxicants is 1.0.	Contaminants are treated; quantitative residual contamination is below clean-up levels.	Reduces risk to the community over the short-term; potentially small exposure to workers; does not cause environmental impacts; the restoration time frame is 12 years.	Biodegradation may not work;, ease of undertaking additional actions is good; ability to monitor is high; other approvals can be obtained; coordination with other agencies is moderate.	3,000,000 $
**Alternative 3** - Pulsed pumping, 7 well points, air-stripping, reinjection, enhanced biodegradation	Regional risk is 10^_ 5^ for carcinogens, and the HI is 1.0.	Contaminants are treated; quantitative residual contamination is below clean-up levels.	Reduces risk to the community over the short-term; potentially small exposure to workers; does not cause environmental impacts; the restoration time frame is 10 years.	Biodegradation may not work; ease of undertaking additional actions is poor; ability to monitor is uncertain because of difficulties in predicting the effect of reinjection; approval of underground injection is questionable; coordination with other agencies is moderate.	5,000,000 $

**Table 4:** Dataset of this case study with a flat structure of balancing criteria, developed by the authors from the hypothetical case study from Appendix A in EPA (1988b) (An upward pointing arrow indicates better performance for higher values, whereas a downward pointing arrow indicates better performance for lower values).

Case A	Balancing criteria				
	
Criteria	Long-term effectiveness and permanence	Reduction of toxicity, mobility or volume through treatment	Short term effectiveness	Implementability	Cost
					
	*C*_1_	*C*_2_	*C*_3_	*C*_4_	*C*_5_

**Measurement scale range**	0–4	0–4	0–4	0–4	$
**Preference direction**	↑	↑	↑	↑	↓
**Alternative 1**	0	0	2	4	500,000
**Alternative 2**	3	3	3	3	3,000,000
**Alternative 3**	3	3	4	1	5,000,000

**Table 5:** Dataset of this case study with a hierarchical structure of balancing criteria, developed by the authors from the hypothetical case study from Appendix A in EPA (1988b) (An upward pointing arrow indicates better performance for higher values, whereas a downward pointing arrow indicates better performance for lower values).

Case B	Balancing criteria							
	
Criteria	Long-term effectiveness and permanence		Reduction of toxicity, mobility or volume through treatment		Short term effectiveness			
			
	Risk for carcinogens	Hazard Index (HI)	Treatment of contaminants	Residual contamination	Risk to the community in the short term	Exposure to workers	Impacts on environment	Restoration time
								
	*SC*_1_	*SC*_2_	*SC*_3_	*SC*_4_	*SC*_5_	*SC*_6_	*SC*_7_	*SC*_8_

**Measurement scale range**	0–4	0–4	0–4	0–4	0–4	0–4	0–4	0–4
**Preference direction**	↓	↓	↑	↓	↓	↓	↓	↓
**Alternative 1**	4	4	0	4	4	0	0	4
**Alternative 2**	1	1	3	0	2	1	0	2
**Alternative 3**	1	1	3	0	2	1	0	0

Case B	Balancing criteria (cont.)					
	
Criteria	Implementability					Cost
		
	Reliability of the measures	Ease of taking additional actions	Ability to monitor	Ability to obtain approvals from other agencies	Coordination problems	Present worth cost
						
	*SC*_9_	*SC*_10_	*SC*_11_	*SC*_12_	*SC*_13_	*SC*_14_

**Measurement scale range**	0–4	0–4	0–4	0–4	0–4	$
**Preference direction**	↑	↑	↑	↑	↓	↓
**Alternative 1**	1	3	3	3	0	500,000
**Alternative 2**	2	2	3	2	2	3,000,000
**Alternative 3**	2	0	1	1	2	5,000,000

**Table 6:** Example of input data used in the stochastic modelling for Alternative 2 in MCDA process 3, together with the original input values and the available range of variability (An upward pointing arrow indicates better performance for higher values, whereas a downward pointing arrow indicates better performance for lower values).

Case A	Balancing criteria				
	
Criteria	Long-term effectiveness and permanence	Reduction of toxicity, mobility, or volume through treatment	Short term effectiveness	Implementability	Cost
					
	*C*_1_	*C*_2_	*C*_3_	*C*_4_	*C*_5_

**Measurement scale range**	0–4	0–4	0–4	0–4	$
**Preference direction**	↑	↑	↑	↑	↓
**Original input data**	3	3	3	3	3,000,000
**Simulation 1**	2	2	3	3	3,500,000
**Simulation 2**	2	3	3	2	3,000,000
**Simulation 3**	3	2	3	2	2,600,000
**Range of variability**	2–3	2–3	2–3	2–3	2,100,000 ≤ *value* ≤ 4.500.000

**Table 7:** Preference indices for PROMETHEE-like outranking procedures in MCDA process 1.

	Alternative 1	Alternative 2	Alternative 3

**Alternative 1**		0.40	0.40
**Alternative 2**	0.60		0.40
**Alternative 3**	0.60	0.20	

**Table 8:** Results of MCDA process 1 and 2.

	MCDA process 1		MCDA process 2	
		
	Net Flow	Ranking	Net Flow	Ranking

**Alternative 2**	0.20	1	0.22	1
**Alternative 3**	0.00	2	− 0.07	2
**Alternative 1**	−0.20	3	− 0.15	3

**Table 9:** Results of MCDA process 3 showing rank acceptability indices and pairwise winning indices (in %) and expected ranking.

	Rank Acceptability Indices				Pairwise Winning Indices				
					
	#1	#2	#3		Alternative 1	Alternative 2	Alternative 3	Expected Ranking	

**Alternative 1**	0.00	0.00	100.00	**Alternative 1**	0.00	0.00	0.00	**Alternative 2**	−105.21
**Alternative 2**	94.79	5.21	0.00	**Alternative 2**	100.00	0.00	94.79	**Alternative 3**	−194.79
**Alternative 3**	5.21	94.79	0.00	**Alternative 3**	100.00	5.21	0.00	**Alternative 1**	−300.00

**Table 10:** Results of MCDA process 4 showing rank acceptability indices and pairwise winning indices (in %) and expected ranking.

	Rank Acceptability Indices				Pairwise Winning Indices				
					
	#1	#2	#3		Alternative 1	Alternative 2	Alternative 3	Expected Ranking	

**Alternative 1**	0.00	16.71	83.29	**Alternative 1**	0.00	0.22	15.94	**Alternative 2**	−108.12
**Alternative 2**	92.10	7.69	0.22	**Alternative 2**	99.77	0.00	91.97	**Alternative 3**	− 207.9
**Alternative 3**	8.03	76.02	15.94	**Alternative 3**	83.52	7.90	0.00	**Alternative 1**	− 283.29

**Table 11:** Results of MCDA process 4 for each balancing criterion, showing RAIs and PWIs (in %).

***C*_1_ :Long-term effectiveness and permanence**							
	**Rank Acceptability Indices**				**Pairwise Winning Indices**		
	**#1**	**#2**	**#3**		**Alternative 1**	**Alternative 2**	**Alternative 3**
**Alternative 1**	0.00	0.00	100.00	**Alternative 1**	0.00	0.00	0.00
**Alternative 2**	68.76	31.24	0.00	**Alternative 2**	100.00	0.00	31.23
**Alternative 3**	68.77	31.23	0.00	**Alternative 3**	100.00	31.24	0.00
***C*_2_ :Reduction of toxicity, mobility or volume through treatment**							
	**Rank Acceptability Indices**				**Pairwise Winning Indices**		
	**#1**	**#2**	**#3**		**Alternative 1**	**Alternative 2**	**Alternative 3**
**Alternative 1**	0.00	0.00	100.00	**Alternative 1**	0.00	0.00	0.00
**Alternative 2**	68.75	31.25	0.00	**Alternative 2**	100.00	0.00	31.24
**Alternative 3**	68.76	31.24	0.00	**Alternative 3**	100.00	31.25	0.00
***C*_3_ :Short term effectiveness**							
	**Rank Acceptability Indices**				**Pairwise Winning Indices**		
	**#1**	**#2**	**#3**		**Alternative 1**	**Alternative 2**	**Alternative 3**
**Alternative 1**	1.95	20.29	77.76	**Alternative 1**	0.00	13.26	0.78
**Alternative 2**	33.22	53.52	13.26	**Alternative 2**	80.88	0.00	23.07
**Alternative 3**	76.93	22.29	0.78	**Alternative 3**	94.93	66.78	0.00
***C*_4_ Implementability**							
	**Rank Acceptability Indices**				**Pairwise Winning Indices**		
	**#1**	**#2**	**#3**		**Alternative 1**	**Alternative 2**	**Alternative 3**
**Alternative 1**	94.19	5.81	0.00	**Alternative 1**	0.00	92.33	100.00
**Alternative 2**	7.67	91.84	0.49	**Alternative 2**	5.81	0.00	99.37
**Alternative 3**	0.00	0.63	99.36	**Alternative 3**	0.00	0.49	0.00
***C*_5_ :Cost**							
	**Rank Acceptability Indices**				**Pairwise Winning Indices**		
	**#1**	**#2**	**#3**		**Alternative 1**	**Alternative 2**	**Alternative 3**
**Alternative 1**	100.00	0.00	0.00	**Alternative 1**	0.00	100.00	100.00
**Alternative 2**	0.00	94.79	5.21	**Alternative 2**	0.00	0.00	94.79
**Alternative 3**	0.00	5.21	94.79	**Alternative 3**	0.00	5.21	0.00

## Footnotes

1 In this research, the cost criterion (*c*_5_) has not been divided into sub-criteria as the hypothetical case study did not use them. However, these metadata are also available for such criterion in the feasibility studies as shown for example in the one of EPA (2016), where the capital, periodic and present worth costs are considered, and in the one of AECOM (2019), which include capital, periodic and annual operational and management costs.
