# Supplementary material for: Supporting contaminated sites management with Multiple Criteria Decision Analysis: Demonstration of a regulation-consistent approach
Source: J Clean Prod. Author manuscript; Available in PMC 2022 Sep 20. (PMC8788621; doi:10.1016/j.jclepro.2021.128347)
Supplement: Supplementary Data [file NIHMS1762309-supplement-Supplementary_Data.docx]

# **ELECTRONIC SUPPLEMENTARY INFORMATION (ESI)**

# Supporting Contaminated Sites Management with Multiple Criteria Decision Analysis: Demonstration of a Regulation-Consistent Approach

Table of Contents

[Appendix 1. Table 1: U.S. EPA employees (stakeholders) contributing their experiences to understanding the context, limitations and main challenges faced during the SRRP’s remediation alternatives prioritization process 2](#_Toc71036858)

[Appendix 2. Methodological details on the selected PROMETHEE-based methods 3](#_Toc71036859)

[Appendix 3. Further details on the selection of the hypothetical case study 6](#_Toc71036860)

[Appendix 4. Why isn’t the “best performer” still the best? 7](#_Toc71036861)

[References 8](#_Toc71036862)

# Appendix 1. Table : U.S. EPA employees (stakeholders) contributing their experiences to understanding the context, limitations and main challenges faced during the SRRP’s remediation alternatives prioritization process

| **Organization/Department** | | **Meeting 1: 18 December 2019** | **Meeting 2: 7 January 2020** | **Meeting 3: 27 February 2020** |
| --- | --- | --- | --- | --- |
| EPA, Office of Research and Development (ORD), Center for Environmental Solutions and Emergency Response (CESER) | Superfund and Technology Liaison (STL) |  | Region 2, Diana Cutt | Region 2, Diana Cutt |
|  | Land Remediation and Technology Division (LRTD)   - Contaminated Sites and Sediments Branch (CSSB) - Emerging Contaminants and Technologies Branch (ECTB) | Robert Ford (CSSB), Director of the Engineering Technical Support Center (ETSC)  John McKernan, Branch Chief (ECTB) | Robert Ford (CSSB), Director of the ETSC  John McKernan, Branch Chief (ECTB) | Robert Ford (CSSB), Director of the ETSC |
| EPA Remedial Project Managers | | Region 1, Carol Keating |  |  |
| EPA Superfund & Emergency Response Division | | Region 7, Lynn Juett, Branch Chief of the Site Remedial Branch | Region 2, Chloe Metz, Remedy Selection, Design & Construction Manager |  |
| EPA Laboratory Services and Applied Science Division, Superfund Support Team (LSASD-SST) | |  | Region 2, Mindy Pensak, Regional Science Liaison |  |
| EPA, Office of Superfund Remediation and Technology Innovation (OSRTI) | National Remedy Review Board (NRRB) |  | Christine Poore, Chair |  |
|  | Science Policy Branch |  | Matthew Lambert, Policy and Technical Support for Superfund |  |

# Appendix 2. Methodological details on the selected PROMETHEE-based methods

PROMETHEE (Brans and Vincke 1985, Behzadian et al. 2010) is a family of MCDA methods among the most well-known and applied in the field of decision analysis. Several PROMETHEE methods have been developed to deal with choice, ranking and sorting problems. Here, we review the PROMETHEE II method and its extensions, considering a hierarchical structure of criteria and robustness recommendations since, in this paper, we are interested in ranking problems with the above structure of criteria and concerns.

Given a set of alternatives $A=\left\{ a,b,\ldots\right\}$ evaluated on $m$ evaluation criteria $G=\left\{ g_{1},\ldots,g_{m} \right\},$ ranking problems consist of rank-ordering all considered alternatives constituting set $A$, from the best to the worst, with the possibility of sharing the ranks by some of them. For the sake of simplicity and without loss of generality, in the following, we shall assume that criteria are expressed on a quantitative scale and that all of them are of the gain type (the greater the evaluation of alternative $a$ on criterion $g_{j}$, that is $g_{j}(a)$, the better $a$ is on the considered criterion). Each criterion is associated with a weight $w_{j}$ representing the relative importance of $g_{j}$ inside the family of criteria $G$. The weights $w_{1}, \ldots,w_{m}$ are such that $w_{j}>0$ for all $g_{j}$ and $\sum_{j=1}^{m} w_{j}=1.$

#### The PROMETHEE II method - MCDA process 1

PROMETHEE II provides a complete ranking of the considered alternatives by means of a procedure composed of the following steps:

1. For each criterion $g_{j}\in G$ and for each ordered pair of alternatives $\left( a,b \right)\in A\times A$ define a partial preference function $P_{j}(a,b)$ being a non-decreasing function of $d_{j}\left( a,b \right)=g_{j}\left( a \right)-g_{j}(b)$. It represents how much $a$ is preferred to $b$ on $g_{j}.$ In Brans and Vincke (1985), six different expressions of $P_{j}(a,b)$ have been defined. Here, we shall use the simplest one:

$P_{j}\left( a,b \right)=\left\{ \begin{matrix} 1 & \mathrm{if} & g_{j}\left( a \right)>g_{j}\left( b \right) \\ 0 & if & g_{j}\left( a \right)\leq g_{j}\left( b \right) \end{matrix} \right.$ $($1$)$

That is, if $a$ is strictly better than $b$ on criterion $g_{j}$, the marginal preference index $P_{j}\left( a,b \right)$ is equal to one. Otherwise, i.e., if $a$ is equal or worse than $b$ on $g_{j}$, $P_{j}\left( a,b \right)=0$.

1. For each $\left( a,b \right)\in A\times A$ compute

$$\pi\left( a,b \right)=\sum_{j=1}^{m} w_{j}P_{j}\left( a,b \right) (2)$$

$\pi\left( a,b \right)\in[0,1]$ and the greater $\pi\left( a,b \right),$ the more $a$ is preferred to $b$. Both $P_{j}(a,b)$ and $\pi(a,b)$ consider the alternatives $a$ and $b$ only, neglecting all other alternatives in $A.$ Note that $\pi\left( a,b \right)$ quantifies a comprehensive advantage of $a$ over $b$ on all criteria while considering their importance coefficients. Thus, $\pi\left( a,b \right)=0.6$ means that the strength of the coalition (i.e., group) of criteria that support the assertion that alternative *a* is strictly better than alternative *b* is 60%.

1. For each $a\in A$ compute the positive, negative and net flows. On the one hand, the positive flow $\phi^{+}(a)$ represents how much, on average, $a$ is preferred to all other alternatives in $A;$ on the other hand, the negative flow $\phi^{-}(a)$ represents how much, on average, all other alternatives in $A$ are preferred to $a$; finally, the net flow $\phi(a)$ is a balance between the two previous flows, and it expresses the comprehensive performance of $a$ in view of the arguments in favor of its strength and weakness. The three indices are computed in the following way:

$$\phi^{+}\left( a \right)=\frac{1}{\left| A \right|-1}\sum_{b\in A\setminus\{a\}} \pi\left( a,b \right); \phi^{-}\left( a \right)=\frac{1}{\left| A \right|-1}\sum_{b\in A\setminus\{a\}} \pi\left( b,a \right); \phi\left( a \right)=\phi^{+}\left( a \right)-\phi^{-}\left( a \right) (3)$$

2. Define a preference relation $P$ and an indifference relation $I$ on $A$ as follows:

$$aPb \mathrm{iff} \phi\left( a \right)>\phi\left( b \right) (4)$$

$$aIb \mathrm{iff} \phi\left( a \right)=\phi\left( b \right) (5)$$

Based on the net flows $\phi(a)$, it is, therefore, possible to compare all alternatives pairwise and, consequently, impose a complete ranking on the set of alternatives.

#### SMAA-PROMETHEE II - MCDA process 3

As already described in the previous section, the PROMETHEE II method is based on the use of deterministic input data. In real-world applications, like in the MCDA process Phases 3 and 4 for remedy alternative assessments, the uncertainty in the evaluation of alternatives can be included in the modeling. In this case, more than one vector of performances can be considered to account for the possible variability in the input. Therefore, to obtain more robust conclusions on the problem under examination, the Stochastic Multicriteria Acceptability Analysis (SMAA) (Lahdelma et al. 1998, Pelissari et al. 2020) has been applied to the PROMETHEE II method by Corrente et al. (2014). SMAA provides robust recommendations considering the plurality of models compatible with the performance of the alternatives and preferences provided by the DM in statistical terms. In particular, the application of SMAA to the PROMETHEE II methods begins from the sampling of several compatible models and the computation of the corresponding alternative’s rankings for each of them. Then, two different indices are computed:

- The *rank acceptability index* (RAI) (Lahdelma et al. 1998), $b_{k}(a)$: is the frequency with which the alternative $a$ reaches the position $k$ in the considered alternative ranking. $b_{k}\left( a \right)\in[0\%,100\%]$, and the best alternatives are the ones presenting high values of $b_{k}(a)$ for the first ranking positions;
- The *pairwise winning index* (PWI) (Leskinen et al. 2006), $p(a,b)$: is the frequency with which alternative $a$ is preferred to alternative $b$. Of course, the greater $p(a,b)$, the more $a$ is preferred to $b$.

To summarize the different RAIs into a complete ranking of the alternatives at hand, several procedures have been proposed by Kadziński and Michalski (2016). One of them, being the one we shall use in this paper, is the expected ranking. It associates a real number to each alternative $a$:

$$ER\left( a,A,RAI \right)=-\sum_{k=1}^{\left| A \right|} k\cdot b_{k}\left( a \right) (6)$$

being a weighted sum of the different RAIs having weights 1, 2, 3, … All the alternatives are ordered in a non-decreasing fashion with respect to the $ER\left( a,A,RAI \right)$ values.

#### The hierarchical PROMETHEE II and hierarchical SMAA-PROMETHEE II - MCDA process 2 and 4.

In some real-world applications, like in the MCDA process 2 and 4, all criteria are not located at the same level. In turn, they are structured hierarchically so that a root criterion can be underlined (being the main objective of the problem), some macro-criteria descend from the root criterion, and so on until reaching the bottom of the hierarchy, where the elementary criteria are placed. The evaluation of the alternatives, as well as the specification of the technical parameters (weights of criteria), are given with respect to these criteria only.

To deal with problems in which criteria are structured hierarchically, the Multiple Criteria Hierarchy Process (MCHP) has been introduced by Corrente et al. (2012). In particular, the extension of the PROMETHEE II method to the MCHP has been presented by Corrente et al. (2013). The application of the MCHP concepts to the PROMETHEE II method permits one to give recommendations not only at a comprehensive level, that is, considering all criteria together, but also partially, considering some particular aspects, may be uniquely important for the remedy alternatives assessment at a certain site. It permits to define a preference and an indifference relation between the alternatives in each node (corresponding to a subset of criteria) of the hierarchy tree (not only at the comprehensive level).

Denoting by $g_{r}$, a generic criterion in the hierarchy, and by $E(g_{r})$, the set of the indices of the elementary criteria descending from $g_{r}$, the generalization of the PROMETHEE II method to the MCHP is based on the following computations:

- the net flow of an alternative $a$ on criterion $g_{r}$, $\phi_{r}(a)$,

$\phi_{r}\left( a \right)=\phi_{r}^{+}\left( a \right)-\phi_{r}^{-}\left( a \right)=\frac{1}{\left| A \right|-1}\sum_{x\in A\setminus\{a\}} \sum_{t\in E\left( g_{r} \right)} w_{t}\cdot P_{t}\left( a,x \right)-\frac{1}{\left| A \right|-1}\sum_{x\in A\setminus\{a\}} \sum_{t\in E\left( g_{r} \right)} w_{t}\cdot P_{t}\left( x,a \right)$ (7)

In the computations of $\phi_{r}(a)$, we consider only the elementary criteria descending from $g_{r}$, neglecting all the others in the hierarchy. Based on $\phi_{r}\left( a \right)$, the ranking of alternatives on criterion $g_{r}$ can be easily computed, providing recommendations at a partial level in addition to the ones obtained at the comprehensive level. The reader interested in more technical details of the reviewed methods is referred to the original papers in which they have been presented.

# Appendix 3. Further details on the selection of the hypothetical case study

From our perspective, the main advantage of using the case study presented in the original US EPA guidance document (EPA 1988) is that it forces the reader to examine the content of the guidance relative to a hypothetical decision process.  While guidance can change over time, it is not common within the US EPA since there is a desire to maintain a consistent starting point for the remedy selection process.  The guidance is the “rule book” by which US EPA remedial project managers (RPMs) structure the Remedial Investigation/Feasibility Study process for any given site. So, when US EPA RPMs see a “case study” presented in the literature, their inclination is to see whether the authors link the presented analysis and recommendations back to the underlying guidance (whether specific to US EPA or guidance implemented in other countries). If the focus is primarily demonstrating performance of the new tool/method for a specific situation, without establishing connection back to the underlying US EPA guidance, then the authors have failed to make a convincing argument for US EPA RPMs.

Our view is that this article is taking a step back to present an approach for developing/selecting the MCDA method linking to the underlying US EPA guidance so that it can be applied with consistency for any particular “case study”.  RPMs are more interested in seeing a consistent approach that provides insight into how to develop/design/select the MCDA method in a way that aligns with the general requirements outlined in US EPA guidance, with tailoring to best suit the decision(s) to be made for a given site.  The chosen “case study” used to illustrate the approach for developing/choosing the MCDA method is not the focus here, as long as the approach can be consistently applied for other “case studies”. The latter is true for our research.

# Appendix 4. Why isn’t the “best performer” still the best?

There might be cases where the stakeholders/DMs think that they have holistic preferences that are “good enough” for a final decision to be taken, without considering the recommendation from an MCDA. We define this as the “the best performer” issue and provide in this appendix some considerations about its risks. This perspective of “best performance” is often based on knowledge of past performance at other sites, which may not replicate site-specific factors (e.g., physical setting) that might degrade performance at the particular site under review. Explicit consideration of site-specific factors that differ relative to sites at which a remediation technology has been used could (and should) be formally included within the MCDA process. Some of this is at a level of detail that is not fully addressed in the balancing criteria, as written. The balancing criteria were written in an attempt to be comprehensive at a high level but inevitably lack detailed specificity. In some cases, our technical knowledge of what does and does not work well has evolved since the publication of the overriding guidance. This presents an opportunity to optimize the MCDA structure/detail to improve the reliability of the selection assessment.

An illustration would be the explicit consideration of unintended consequences from use of a remediation technology that has been shown to perform well for treatment of a particular contaminant. In-situ chemical oxidation (ISCO) is a treatment technology that has been demonstrated to perform well to destroy certain organic contaminants within a groundwater plume. However, this technology induces two conditions that may not naturally exist in the aquifer: 1) acidic pH and 2) strongly oxidizing conditions. Both induced conditions can result in mobilization of naturally occurring elements in the aquifer matrix: 1) arsenic under very acidic pH in the treatment zone, and 2) selenium/uranium under strongly oxidizing conditions in the treatment zone (Gardner et al. 2015). The degree to which this becomes a problem will depend on the local geology, which dictates the relative concentrations of these elements that might be encountered. By not explicitly considering these unintended consequences in relation to the potential to degrade groundwater quality at the expense of treating the organic contaminant, the selection process has uncertainty that might actually downgrade the technical value of decision to select the ISCO technology. It is only within the past 5-years that this technical knowledge has been more generally recognized. Likewise, specific technical issues like this are not explicitly listed in a comprehensive fashion in the balancing criteria. There may be general acknowledgement of the technical issue within these criteria, but one would have to deliberately incorporate these factors in the MCDA process to provide a fair and comprehensive evaluation. One would have to fairly consider the uncertainty of the existing site characterization information, added complexity of the engineering design and/or implementation cost to address the unintended consequences that might be associated with what is otherwise a remediation technology that performs well for destruction of the primary contaminant of interest (Baciocchi et al. 2014, Pac et al. 2019).

# References

Baciocchi, R., L. D'Aprile, I. Innocenti, F. Massetti, and I. Verginelli. 2014. Development of technical guidelines for the application of in-situ chemical oxidation to groundwater remediation. Journal of Cleaner Production **77**:47-55.

Behzadian, M., R. B. Kazemzadeh, A. Albadvi, and M. Aghdasi. 2010. PROMETHEE: A comprehensive literature review on methodologies and applications. European Journal of Operational Research **200**:198-215.

Brans, J. P., and P. Vincke. 1985. A Preference Ranking Organisation Method. The PROMETHEE method for MCDM. Management Science **31**:647-656.

Corrente, S., J. R. Figueira, and S. Greco. 2014. The SMAA-PROMETHEE method. European Journal of Operational Research **239**:514-522.

Corrente, S., S. Greco, and R. Słowiński. 2012. Multiple Criteria Hierarchy Process in Robust Ordinal Regression. Decision Support Systems **53**:660-674.

Corrente, S., S. Greco, and R. Słowiński. 2013. Multiple Criteria Hierarchy Process with ELECTRE and PROMETHEE. Omega **41**:820-846.

EPA, U. S. 1988. Guidance on Remedial Actions for Contaminated Ground Water at Superfund Sites. U.S. Environmental Protection Agency, EPA/540/G-88/003.

Gardner, K., E. Hadnagy, S. Greenwood, B. Smith, R. Fimmen, D. K. Nair, and H. V. Rectanus. 2015. Final Report: Impacts on Groundwater Quality Following the Application of ISCO: Understanding the Cause of and Designing Mitigation for Metals Mobilization. SERDP Project ER-2132. Accessed on 31 May 2021 at: <https://www.serdp-estcp.org/Program-Areas/Environmental-Restoration/Contaminated-Groundwater/Emerging-Issues/ER-2132>.

Kadziński, M., and M. Michalski. 2016. Scoring procedures for multiple criteria decision aiding with robust and stochastic ordinal regression. Computers & Operations Research **71**:54-70.

Lahdelma, R., J. Hokkanen, and P. Salminen. 1998. SMAA - Stochastic multiobjective acceptability analysis. European Journal of Operational Research **106**:137-143.

Leskinen, P., J. Viitanen, A. Kangas, and J. Kangas. 2006. Alternatives to Incorporate Uncertainty and Risk Attitude in Multicriteria Evaluation of Forest Plans. Forest Science **52**:304-312.

Pac, T. J., J. Baldock, B. Brodie, J. Byrd, B. Gil, K. A. Morris, D. Nelson, J. Parikh, P. Santos, M. Singer, and A. Thomas. 2019. In situ chemical oxidation: Lessons learned at multiple sites. Remediation Journal **29**:75-91.

Pelissari, R., M. C. Oliveira, S. B. Amor, A. Kandakoglu, and A. L. Helleno. 2020. SMAA methods and their applications: a literature review and future research directions. Annals of Operations Research, pages 1–61. DOI: 10.1007/s10479-019-03151-z.
